# Thiopurine methyltransferase (TPMT) genotyping to predict myelosuppression risk

**DOI:** 10.1371/currents.RRN1236

**Published:** 2011-05-15

**Authors:** Christine M. Nguyen, Margaret A.S. Mendes, Joseph D. Ma

**Affiliations:** ^*^VA San Diego Healthcare System and ^‡^UCSD Skaggs School of Pharmacy and Pharmaceutical Sciences

## Abstract

Azathioprine (AZA), 6-mercaptopurine (6-MP), and thioguanine (TG) are thiopurine drugs. These agents are indicated for the treatment of various diseases including hematologic malignancies, inflammatory bowel disease (IBD), rheumatoid arthritis, and as immunosuppressants in solid organ transplants. Thiopurine drugs are metabolized, in part, by thiopurine methyltransferase (TPMT). TPMT displays genetic polymorphism resulting in null or decreased enzyme activity. At least 20 polymorphisms have been identified, of which, TPMT *2, *3A, *3B, *3C, and *4 are the most commonly studied. These polymorphisms have been associated with increased myelosuppression risk. TPMT genotyping may be useful to predict this risk.

## **Clinical Scenario**

Approximately 86-97% of patients have the TPMT*1/*1 (wild-type) genotype.[1]  These patients have normal *TPMT* enzyme activity.  Approximately 3-14% of patients have the heterozygous *TPMT* genotype and possess one *TPMT* variant allele.  These patients may experience moderate to severe myelosuppression; therefore, thiopurine drug dose reduction may be warranted.[1] [2]  The population prevalence of patients who have the homozygous *TPMT* variant genotype is low (approximately 1 in 178 to 1 in 3,736 patients).[1]  However, these patients have the highest risk of developing severe myelosuppression, which may lead to life-threatening complications such as sepsis.[1] [2] [3]  These patients may not be candidates for treatment with a thiopurine drug, or the drug dose should be reduced by at least 10-fold.[1]

## **Test Description**

The *TPMT* genotype assay uses polymerase chain reaction (PCR) amplication followed by single nucleotide primer extension to detect the TPMT*1, *2, *3A, *3B, and/or *3C alleles.[4] [5]  The *TPMT* genotype assay requires a whole blood sample.

## **Public Health Importance**

Thiopurine-induced myelosuppression can result in increased morbidity, hospitalization and/or treatment discontinuation.[6] [7]  Myelosuppression increases an individual’s risk of developing an infection and sepsis.[8] [9] [10] [11]  The incidence of mild leukopenia is approximately 5-25%.[12]  Rare, but severe leukopenia can develop suddenly and unpredictably in approximately 3% of patients.[13]  A 27-year analysis showed that AZA contributed to the incidences of myelosuppression in 5% of patients.[10]  Over an 18-year period, 2% of patients with IBD experienced 6-MP-induced leukopenia that resulted in hospitalization.[14]  The incidence of myelosuppression occurred more frequently during the first eight weeks after treatment initiation, and was more likely to occur with a higher drug dose.[14] [15]  It should be noted that while the *TPMT* genotype test may predict myelosuppression risk, the test should not replace complete blood count (CBC) monitoring to detect myelosuppression during treatment with thiopurine drugs.[2] [16]  Given that TG carries the risk of myelosuppression[17], CBC monitoring is also necessary for this drug.  

## **Published Reviews, Recommendations and Guidelines** 

**Systematic evidence reviews**

The Agency for Healthcare Research and Quality (AHRQ) concluded that “there is currently insufficient evidence regarding the effectiveness of determining *TPMT* status prior to thiopurine treatment in terms of improvement in clinical outcomes and incident myelotoxicity in comparison with routine monitoring of full blood counts and adverse events."[2]

**Recommendations by independent groups**

- The Clinical Pharmacogenomics Implementation Consortium (CPIC) guidelines:[1]
  - Heterozygous *TPMT* genotype (intermediate activity): In patients who possess a single *TPMT* functional (*1) and nonfunctional allele (*2, *3A, *3B, *3C, or *4), the initial dose of AZA or 6-MP should be reduced by 30-70%.  The AZA dose can be titrated as tolerated.  The 6-MP dose should be adjusted based on the severity of myelosuppression and disease-specific guidelines.  The initial dose of TG should be reduced by 30-50%, and adjusted based on the severity of myelosuppression and disease-specific guidelines.
  - Homozygous *TPMT* genotype (variant mutant, low, or deficient activity): Reduce the initial dose of AZA, 6-MP or TG by 10-fold and extend the dosing frequency from daily to three times weekly, or select an alternative drug.
- The Royal Dutch Association for the Advancement of Pharmacy Pharmacogenomic Working Group:[18]
  - Patients who are intermediate metabolizers based on the *TPMT* genotype or phenotype test: The dose of AZA or 6-MP should be reduced by 50% and titrated based on hematologic monitoring and efficacy, or select an alternative drug. 
  - Patients who are poor metabolizers based on the *TPMT* genotype or phenotype test: The dose of AZA or 6-MP should be reduced by 90% and titrated based on hematologic monitoring and efficacy, or select an alternative drug.
  - Patients who are intermediate or poor metabolizers should not be treated with TG as there are “insufficient data to allow calculation of dose adjustment.”

**Guidelines by professional groups**

- The American College of Gastroenterology treatment guidelines prefer *TPMT* phenotyping over genotyping because the phenotype assay quantifies the level of *TPMT* enzyme activity in patients who are being treated with thiopurines for ulcerative colitis (UC).[16]
- The British Society of Gastroenterology does not require either *TPMT* genotyping or phenotyping as a prerequisite to initiating thiopurine therapy because the use of AZA has been shown to be safe in patients with Crohn’s disease (CD) or UC.[13] [19]    

**Other groups**

- The US Food and Drug Administration (FDA) and prescribing information for AZA and 6-MP recommend either *TPMT* genotyping or phenotyping prior to initiating therapy to help identify patients who are at an increased risk of developing toxicity.[20] [21] [22]
- The prescribing information for TG indicates that patients with *TPMT* deficiency “may be unusually sensitive to the myelosuppressive effects of TG.  Substantial dosage reductions may be required to avoid the development of life-threatening bone marrow suppression.  Prescribers should be aware that some laboratories offer testing for *TPMT* deficiency.”[17]  Despite the risk of myelosuppression and the availability of *TPMT* tests, the manufacturer does not indicate that testing for *TPMT* deficiency prior to initiating TG is recommended, or provide dose adjustment guidance based on the *TPMT* test result.

## **Evidence Overview** 

***Analytic Validity***

      A systematic review of published literature was conducted to determine the characteristics of the *TPMT* tests.[23]  The literature evaluated *TPMT* tests in patients with various ethnicities (e.g., Caucasians, Asians, and African Americans).  The patient population analyzed was non-specific; healthy volunteers, those with medical conditions that require thiopurine (e.g., acute lymphoblastic leukemia, IBD), children, and adults were included.  The results showed that there are eight different ways in which the *TPMT* genotype tests can be performed, and no single "gold standard" method for comparison has been accepted.  Overall, the *TPMT* genotype tests had a lower sensitivity and a higher positive predictive value (PPV) in comparison to the *TPMT* phenotype test.  Furthermore, there was a wide range of sensitivity, specificity, PPV and negative predictive value (NPV), which could be due to the variety of genotyping methods.  Additionally, these ranges were derived from evaluations of genotype testing methods compared with various reference standards that included both genotyping and enzymatic tests.

- Sensitivity: 55-100%
- Specificity: 94-100%
- PPV: 67-100%
- NPV: 76-100%

***Clinical Validity***

- Patients with homozygous *TPMT* (inactive) genotype who are treated with conventional doses of thiopurines have 100% likelihood of developing severe myelosuppression.[1]  The odds ratio (OR) of developing leukopenia is 18.60 (95% CI = 4.12-83.60).[2]
- Approximately 30-60% of patients with heterozygous *TPMT* genotype experience severe myelosuppression when standard doses of thiopurines are administered.[1]  The OR of developing leukopenia is 4.62 (95% CI = 2.34-9.16).[2]
- Approximately 6-11% of Caucasians have the heterozygous *TPMT* genotype, and 0.03% have the homozygous variant *TPMT* genotype.[24] [25] [26] [27]  Southeast Asians and Japanese have a lower rate of the heterozygous *TPMT* genotype (1.2-2%) and homozygous variant *TPMT* genotype (<1%).[28] [29]
- Concordance between *TPMT* genotype and phenotype vary in the published literature.  One study showed that approximately 43% of IBD patients who possess one *TPMT* variant allele had increased *TPMT* enzyme activity.[30]  In another study, the correlation between *TPMT* phenotype and genotype was 99% amongst patients with IBD who were treated with AZA.[31]

***Clinical Utility***

- No studies were identified that directly address clinical utility in regards to improving health outcomes (e.g., mobidity and mortality).
- Although patients with the *TPMT* wild-type genotype are less likely to experience leukopenia, some patients may experience this adverse event at a rate ranging from 8% to 43%, which leads to either dose reduction or treatment discontinuation.[28] [30] [32] [33] [34] [35]  Conversely, patients with the heterozygous *TPMT* genotype may not experience myelosuppression at all.[32] [34] [36]
- Approximately 71-91% of patients with the heterozygous *TPMT* genotype do not experience severe leukopenia, and 25% of patients with the homozygous variant *TPMT* genotype do not experience severe leukopenia.[32] [34]
- The *TPMT* genotype test is designed to detect a limited number of mutations.  Therefore, it cannot be considered the gold standard to determine *TPMT* activity.[23]
- From a third-party payer’s perspective, a one-year cost-effectiveness analysis reported that implementing the *TPMT* genotyping is cost-effective versus standard of care in patients with chronically active steroid-treated CD.[37]  Patients achieved a quicker response time compared to standard of care (19.19 weeks vs. 22.41 weeks).

***Clinical Considerations***

      *TPMT* genotype status is not the sole reason for increased myelosuppression risk in patients who are treated with thiopurine drugs.  Myelosupprression may be the result of drug-drug interactions with agents such as, but not limited to, allopurinol, mesalamine, metronizadole, non-steroidal anti-inflammatory drugs (NSAIDs), and sulfamethoxazole/trimethoprim.[24]

## **Links**

- Pharmacogenomics Knowledge Base (PharmKB): Azathioprine
- Agency for Healthcare Research Quality (AHRQ): Thiopurine Methyltransferase Activity 
- US Food and Drug Administration: Table of Pharmacogenomic Biomarkers in Drug Labels 

## **Funding information**

Christine M. Nguyen and Margaret A.S. Mendes are funded by the Veterans Affairs San Diego Healthcare System. Joseph D. Ma is funded by the University of California, San Diego Skaggs School of Pharmacy and Pharmaceutical Sciences.   

## **Competing interests**

The authors have declared that no competing interests exist. 
